# Effects of dietary defatted olive seed flour supplementation on growth performance, systemic status, and intestinal health in broiler chickens

**DOI:** 10.3389/fvets.2026.2002766

**Published:** 2026-10-21

**Authors:** Ibrahim A. Alhidary, Abdulrahman S. Alharthi, Yasser A. Alayedh, Hani H. Al-Baadani

**Affiliations:** Department of Animal Production, College of Food and Agriculture Science, King Saud University, Riyadh, Saudi Arabia

**Keywords:** biochemical profile, broiler performance, defatted olive seed flour, intestinal health, oxidative status

## Abstract

High-growth broilers are prone to gut dysbiosis, oxidative stress, and inflammation. Replacing antibiotic growth promoters with polyphenol- and fiber-rich defatted olive seed flour is a promising strategy. Therefore, this study aimed to evaluate the effects of dietary defatted olive seed flour supplementation on growth performance, carcass traits, serum biochemical profiles, systemic oxidative status, intestinal mucosal cytokine gene expression, the abundance of selected cecal bacterial DNA targets, and ileal histomorphology in broilers. A total of 840 one-day-old male broiler chicks were randomly allocated to five dietary groups (12 replicate pens/group, 14 birds/pen) and received basal mash diets supplemented with 0 (CON), 1 (T1), 2 (T2), 3 (T3), or 4 (T4) g defatted olive seed flour/kg diet for 28 days. Dietary inclusion of defatted olive seed flour (T1–T4) increased final body weight, weight gain, and the production efficiency index and improved feed conversion efficiency (*p* < 0.05); T3 and T4 increased carcass weight and dressing yield (*p* < 0.05). Serum triglycerides and low-density lipoprotein cholesterol were lower at T1 and T2, and total antioxidant capacity was higher and malondialdehyde was lower at T2–T4 (*p* < 0.05). Real-time PCR showed higher cecal *Lactobacillus* spp. and lower *Clostridium* spp., *Escherichia coli*, and *Salmonella* spp. DNA copy numbers with supplementation (*p* < 0.05). Dietary inclusion at 3 g/kg was associated with greater ileal villus height and villus surface area, along with local transcriptional modulation, characterized by higher *IL-10* and *IFN-*γ transcript abundance. In conclusion, responses across the measured endpoints were generally favorable at 2–3 g/kg, although no single inclusion level consistently improved all outcomes under the conditions of this 28-day study.

## Highlights

- Defatted olive seed flour supplementation was associated with improved growth performance without affecting feed intake over the 28-day period.
- Diets containing 2–4 g/kg defatted olive seed flour increased total antioxidant capacity and reduced malondialdehyde.
- Supplementation at 3 g/kg increased ileal villus height and surface area, upregulated *IL-10* and *IFN-*γ expression, and suppressed *IL-6* and *TNF-α*.
- Defatted olive seed flour increased targeted cecal *Lactobacillus* spp. while decreasing *Clostridium* spp., *Escherichia coli*, and *Salmonella* spp. DNA copy numbers.

## Introduction

The modern poultry industry faces a dual challenge: maximizing growth performance and meat yield in high-performing broiler strains while reducing routine reliance on in-feed antibiotic growth promoters (1, 2). Intensive selection for rapid growth in modern broilers increases metabolic demand, making birds highly susceptible to environmental, nutritional, and microbial stressors (3). These stressors trigger gut dysbiosis and damage the intestinal mucosal epithelium, disrupting barrier function, and increasing systemic oxidative stress through excessive generation of reactive oxygen species (4, 5). Sustainable feed additives such as phytogenics, prebiotics, and agro-industrial by-products are therefore needed to strengthen antioxidant defenses, maintain mucosal architecture, and support immune status (6, 7).

Olive (*Olea europaea L*.) cultivation and industrial olive oil extraction generate substantial quantities of solid by-products, including olive pomace, olive cake, olive leaves, and olive seed meal (8). Historically regarded as environmental liabilities, these secondary streams are rich in natural bioactive compounds suitable for monogastric animal nutrition (9). Olive by-products contain functional polyphenols and lipophilic bioactives–primarily oleuropein, squalene, and hydroxytyrosol–along with variable levels of crude fiber (10).

A growing body of literature highlights the biological efficacy of olive-derived materials in poultry diets. For instance, dietary inclusion of olive pomace extracts has been shown to improve growth performance, enhance intestinal integrity, and positively modulate gut bacterial populations in broilers (11, 12). Similarly, supplementing broiler diets with olive pomace and bioactive extracts increased total antioxidant capacity and reduced systemic lipid peroxidation in another study (13). Fermented or enzymatically treated olive pomace formulations have also been reported to upregulate digestive enzyme expression, improve glucose transporter expression, and enhance meat oxidative stability during frozen storage (14). Furthermore, olive leaf powder supplementation increased circulating immunoglobulins, enhanced beneficial cecal bacterial populations, and improved growth efficiency in broilers (15).

Despite these promising insights, the practical use of conventional, raw olive by-products (e.g., crude olive pomace, olive cake, or raw leaf meals) in broiler diets is often limited by their high physical variability, elevated residual oil content, and high proportions of poorly digestible fiber. Defatted olive seed flour differs from these unrefined co-products. Produced specifically from destoned olive seeds by mechanical cold pressing followed by solvent extraction, this processing removes excess triacylglycerols to yield a standardized matrix with a low residual fat content (~7.19%) while concentrating the functional crude fiber fraction (~30.0%) (16). Unlike raw pomace, which contains high lipid loads susceptible to rancidity, this defatting process lowers the lipid load and may therefore limit rancidity, while the flour retains the phenolic and terpene compounds detected by gas chromatography–mass spectrometry (GC–MS). GC–MS profiling shows that defatted olive seed flour retains a unique bioactive matrix dominated by oleic acid (82.93%), along with key phenolics and terpenes, including oleuropein (0.49%), squalene (0.47%), and hydroxytyrosol (0.13%) (17). Hydroxytyrosol and oleuropein act as potent free-radical scavengers and have been reported to activate nuclear factor erythroid 2-related factor 2 (Nrf2)-related antioxidant pathways (18). Squalene and the insoluble and soluble fiber fractions, in turn, support mucosal structural stability and serve as fermentable substrates for beneficial lactic acid–producing bacteria in broilers (19).

Previous studies have evaluated raw olive pomace or crude leaf meals (20, 21), but the physiological impacts of defatted olive seed flour across a systematic dietary inclusion gradient remain uninvestigated in healthy broilers. Although recent work has evaluated defatted olive seed flour under an *Eimeria tenella* infection model (17), its dose-dependent baseline effects in unchallenged commercial broilers have not yet been established. This study therefore provides a comprehensive evaluation of defatted olive seed flour across an expanded inclusion gradient (1–4 g/kg feed), assessing growth performance and carcass yields, gut histomorphology, selected bacterial DNA targets, systemic antioxidant status, and mucosal immune expression to determine baseline responses in healthy broilers.

We hypothesized that dietary supplementation with defatted olive seed flour (1–4 g/kg feed) would produce dose-dependent changes in cecal bacterial targets, antioxidant status, and ileal cytokine transcripts in broilers without inducing organ toxicity. Specifically, we posited that the bioactive compounds and fiber fractions in defatted olive seed flour would promote beneficial cecal bacterial targets, reduce systemic lipid peroxidation, and enhance ileal mucosal architecture and a more anti-inflammatory cytokine transcript profile, with corresponding improvements in growth performance and carcass traits. Accordingly, this study aimed to evaluate the dose-dependent effects of defatted olive seed flour (0, 1, 2, 3, and 4 g/kg feed) on growth efficiency, carcass yield, systemic oxidative and metabolic status, absolute abundance of selected cecal bacterial DNA targets, mucosal cytokine transcript profiles, and ileal histomorphology in Ross 308 broilers during a 28-day rearing period.

## Materials and methods

### Ethical statement

All trial methodologies were formally approved before execution by King Saud University’s Research Ethics Committee (Approval No. KSU-SE-24-56; issued October 21, 2024).

### Broilers, housing, and experimental design

A commercial hatchery supplied 840 one-day-old male Ross 308 broilers, which were shipped to the trial facility in insulated, ventilated cardboard boxes at a density of 50 chicks per box. Environmental conditions during transit were maintained at 35.5 °C and 60% relative humidity in temperature-monitored vehicles. On arrival, broiler chicks were sexed by the feather-sexing method, individually weighed, and housed on the floor in an environmentally controlled room for the full 28-day trial. Room temperature was set at 35 °C and relative humidity at 55% at placement, and both were gradually reduced to 20 °C and 40%, respectively, by 22 days of age. A photoperiod of 24 h light (35 lux) was applied during the starter stage from 1 to 14 days of age and then reduced to 18 h light (10 lux) and 6 h dark during the grower stage from 15 to 28 days of age, consistent with the Ross 308 management guide (Aviagen, 2018, New York, NY, USA), which also governed all other environmental settings. The basal mash diet and water were offered *ad libitum* throughout the trial. All chicks were vaccinated by nasal instillation against infectious bursal disease at 1, 7, and 14 days of age, following standard Saudi commercial vaccination protocols. Broiler chicks were allocated using a completely randomized design to five dietary groups, with 12 replicate floor pens per dietary group (experimental units) and 14 birds per pen (168 broilers per dietary group). To ensure uniform weight distribution across all experimental units at placement, chicks were individually weighed and grouped into five weight classes. Within each class, chicks were randomly assigned to floor pens using SAS (PROC PLAN, SAS Institute Inc.) (22), so that the initial average body weight was identical in each pen. Finally, the five dietary treatments were randomly assigned to the 60 floor pens by lot drawing.

### Dietary groups and feed mixing

Diets based on a corn–soybean meal matrix were formulated according to Ross 308 commercial nutrient specifications (Aviagen, 2022, New York, NY, USA) and administered in two sequential feeding phases in mash form: starter (days 1–14) and grower (days 15–28). The ingredient and calculated nutrient composition of both phases of the basal diet (CON group) are presented in Table 1. The five dietary groups were: CON, basal diet without supplementation; and T1, T2, T3, and T4, the basal diet supplemented with 1, 2, 3, and 4 g of defatted olive seed flour/kg feed, respectively.

**Table 1:** Ingredient composition and calculated nutrient analysis of experimental starter and grower basal diets (CON group).

Ingredients (% as feed)^a^	Dietary stages	
	Starter from 1 to 14 days of age	Grower from 15 to 28 days of age
Corn	53.28	53.61
Soybean meal (48% CP)	38.34	37.49
Soy oil	3.57	4.94
Monocalcium phosphate	1.68	1.40
Limestone	1.60	1.39
DL-Methionine	0.41	0.33
L-Lysine HCl	0.30	0.14
L-Threonine	0.18	0.10
Common salt	0.40	0.40
Vitamin premix^b^	0.10	0.10
Mineral premix^c^	0.10	0.10
Choline Cl (60%)	0.04	0.00
Total	100.00	100.00
Calculated nutrient analysis (% as dry matter)		
Dry matter	88.97	89.02
Metabolizable energy (kcal/kg)	3000.00	3100.46
Crude protein	23.00	22.00
Crude fat	6.30	7.66
Crude fiber	2.54	2.51
Calcium	1.00	0.87
Available phosphorus	0.50	0.43
Sodium	0.18	0.16
Chloride	0.29	0.26
Lysine	1.32	1.18
Methionine	0.70	0.62
Total sulfur amino acids	0.98	0.90
Threonine	0.88	0.79

^a^Defatted olive seed flour (0, 1, 2, 3, or 4 g/kg) was added on top of the basal formulation shown above as a feed additive. Based on the analyzed composition of defatted olive seed flour (96.01% DM, 2.65% CP, 7.19% fat, 30.00% crude fiber, and 1.72% ash), inclusion at 1, 2, 3, and 4 g/kg resulted in calculated incremental nutrient shifts of +0.03%, +0.06%, +0.09%, and +0.12% for crude fiber, and +0.007%, +0.014%, +0.021%, and +0.029% for crude fat across T1–T4, respectively. Variations in metabolizable energy and crude protein across diets were <0.05% and <0.01%, respectively, confirming that all experimental diets were practically isocaloric and isonitrogenous. ^b^Vitamin analysis per kg of premix (KAPREVIT, CAP4000213, Al-Wesam, KSA): Vitamin A = 12,000,000 IU, Vitamin D_3_ = 5,000,000 IU, Vitamin E = 80,000 mg, Vitamin K_3_ = 3,200 mg, Vitamin B_1_ = 3,200 mg, Vitamin B_2_ = 8,600 mg, Vitamin B_6_ = 4,300 mg, Vitamin B1_2_ = 17,000 mcg, Vitamin B_5_ = 20,000 mg, Vitamin B_9_ = 2,200 mg, Vitamin B_3_ = 65,000 mg, and Biotin = 220,000 μg. ^c^Mineral analysis per kg of premix (KAPREMIN, CAP4000214, Al-Wesam, KSA): Iron = 20,000 mg, Copper = 16,000 mg, Zinc = 110,000 mg, Manganese = 120,000 mg, Iodine = 1,250 mg, Selenium = 300 mg, Cobalt = 100 mg.

The defatted olive seed flour used in this trial was supplied by Adimax, Adinature S.L. (Spain). It was produced from destoned olive seed by mechanical cold pressing followed by solvent extraction with n-hexane to remove most of the triacylglycerols, yielding a residual crude fat content of 7.19% while concentrating the fiber and bioactive phenolic fraction of the raw material. Based on the manufacturer’s certified composition data (Adimax, Adinature, Spain), the defatted olive seed flour contained 96.01% dry matter, with crude fractions of 2.65% protein, 7.19% fat, 30.00% fiber, and 1.72% ash. Furthermore, the bioactive compound profile of this batch of defatted olive seed flour was previously determined in our laboratory using GC–MS, as shown in Supplementary Table S1. Results are expressed semi-quantitatively as relative area percentages (%) calculated by dividing individual peak areas by the total integrated chromatogram area, indicating relative compound proportions rather than absolute concentrations. GC–MS profiling showed that defatted olive seed flour retains a distinctive bioactive matrix dominated by oleic acid (82.93% relative area), along with key secondary metabolites including oleuropein (0.49%), squalene (0.47%), and hydroxytyrosol (0.13%). Dietary supplementation began on day 1 and continued throughout the 28-day feeding period. Defatted olive seed flour was added to the basal diet (as an additive to the basal diet formulation without replacing major feed ingredients such as corn or soybean meal) by first blending the designated amounts of defatted olive seed flour (1, 2, 3, and 4 g/kg of diet) into a premix with a small portion of the basal mash feed (CON group) using a stepwise vertical-to-horizontal mechanical premixer for 10 min. This concentrated premix was then incorporated into the main batch of the basal mash diet using a twin-shaft ribbon mixer (Model MX-500, Parma, Italy) and mixed for an additional 5 min to ensure uniform distribution. Diet mixing uniformity and batch homogeneity were verified before trial initiation by evaluating microtracer recovery (Coarse Red Micro-Tracer, Microtracer Inc., San Francisco, CA, USA) across 10 random samples per batch, resulting in a coefficient of variation (CV) of <4.2%. Given the low inclusion rates (0.1–0.4% of total diet weight), all experimental diets can be considered practically isonitrogenous and isocaloric, and defatted olive seed flour was omitted from the calculated nutrient values because its diluting effect was negligible. The four supplementation levels (1–4 g/kg) were selected based on preliminary pilot work and previous literature on olive by-products, which indicated that this range balances the prebiotic-like and antioxidant benefits of the fiber (17).

### Growth performance

Average body weight was recorded per replicate floor pen on the initial day and at the end of the starter (14 days of age) and grower (28 days of age) stages. Weight gain for each stage, and for the overall period from 1 to 28 days of age, was calculated by subtracting the initial from the final body weight for the corresponding interval (23). Feed offered and feed refused were recorded for each floor pen at the same 14-day intervals and cumulatively for the whole trial to calculate feed intake. Feed conversion ratio was calculated as total feed intake divided by total body weight gain (23). The European Production Efficiency Index was calculated for each stage as [(livability, % × final body weight, kg)/(feed conversion ratio × age in days)] × 100, as described by Martins et al. (24). The floor pen (replicate floor pen) was used as the experimental unit for all growth performance variables.

### Slaughter procedure and carcass traits

At 28 days of age, two broilers per floor pen (*n* = 24 total broilers per dietary group) were randomly selected, individually weighed to obtain slaughter weight, and processed, and the data from the two sampled birds were subsequently averaged to represent the experimental unit (*n* = 12 pens per group). Carcass dressing percentage was determined by expressing eviscerated carcass weight as a proportion of live preslaughter weight (25). Similarly, primary cuts (breast and leg portions) and key internal and lymphoid organs (liver, intestine, thymus, bursa, and spleen) were recorded and normalized to total slaughter weight following the procedures outlined by Wegner et al. (25). Breast muscle physical traits, including pH and instrumental color coordinates, were measured in duplicate on the pectoralis major muscle at 1 h post-slaughter according to standard meat quality assessment procedures (25, 26). Meat pH was determined using a portable digital pH meter equipped with a spear-type glass electrode (Model HI98163, Hanna Instruments, Padova, Italy) calibrated with pH 4.0 and 7.0 buffer solutions. Instrumental color space values (lightness, redness, and yellowness) were recorded using a handheld chromameter (Model CR-400, Konica Minolta, Tokyo, Japan) calibrated against a standard white tile before measurements and operated with an 8 mm aperture, a 2° standard observer, and a CIE Illuminant C.

### Blood sampling and serum biochemical and oxidative analyses

At 28 days of age, two broilers per floor pen (*n* = 24 total broilers per dietary group) were randomly selected for blood sampling, and their serum parameters were averaged to represent the experimental unit (*n* = 12 pens per group). Approximately 3 mL of blood was drawn from the wing vein into plain (non-anticoagulant) tubes. Serum was separated by centrifugation (Hettich Zentrifugen, Mikro 200R, Germany) at 3,500 × g for 15 min and stored at −80 °C until analysis. Colorimetric measurements of serum metabolites, including total protein, glucose, albumin, total cholesterol, triglycerides, HDL cholesterol, LDL cholesterol, uric acid, creatinine, and aspartate aminotransferase, were performed on an automated clinical chemistry platform (Chem Well, FL, USA) using commercial diagnostic kits (Applied, Amposta, Spain) according to the manufacturer’s instructions. All automated chemistry assays demonstrated intra-assay and inter-assay coefficients of variation (CVs) of <3.2% and <4.8%, respectively. Serum globulin levels were calculated by subtracting albumin from total protein values (27). Total antioxidant capacity (ferric reducing/antioxidant power principle) and malondialdehyde (thiobarbituric acid reactive substances principle), reflecting nonenzymatic antioxidant defense and lipid peroxidation, respectively, were quantified in serum using commercial ELISA kits (Solarbio Life Sciences, China) read on a microplate reader (Bio-Medical, Shenzhen, China) at 593 nm (TAC) and 532 nm (MDA), according to the manufacturers’ instructions. Optical densities were calibrated against standard curve concentrations (*R*^2^ > 0.995), with intra- and inter-assay CVs maintained below 4.5 and 5.5%, respectively.

### Absolute cecal selected bacterial DNA target estimate

After slaughter (28 days of age), cecal digesta (approximately 1 g) were collected from two broilers per floor pen (*n* = 24 broilers per dietary group), pooled per pen, and analyzed with the pen serving as the experimental unit (*n* = 12 pens per group). Samples were immediately flash-frozen in liquid nitrogen and stored at −80 °C until processing. Genomic DNA was isolated using a specialized stool DNA extraction kit (Qiagen, Germantown, MD, USA) according to the manufacturer’s standardized protocol. DNA yield and purity were verified spectrophotometrically (NanoDrop 2000, Thermo, USA) by evaluating A_260_/A_280_ absorbance ratios (acceptable range: 1.80–2.00) before normalizing all samples to 100 ng/μL.

Quantitative real-time PCR (Applied Biosystems, 7,300, MA, USA) was performed in triplicate using SYBR Green chemistry (PCR5505, Haven Scientific, KAUST, SA) and the primer sequences listed in Supplementary Table S2. Thermal cycling conditions consisted of an initial denaturation at 95 °C for 10 min, followed by 40 amplification cycles of 95 °C for 15 s, target-specific annealing at 58–60 °C for 30 s, and extension at 72 °C for 30 s. Five specific bacterial taxa were selected to evaluate the impact of dietary groups on gut health: *Lactobacillus* spp. and *Bifidobacterium* spp. were targeted as key beneficial bacteria that enhance short-chain fatty acid production, maintain mucosal barrier integrity, and promote competitive exclusion, whereas *Salmonella* spp., *Clostridium* spp., and *Escherichia coli* were chosen as representative potentially harmful or opportunistic pathogens that impair nutrient absorption and cause intestinal inflammation.

Primer specificity was first evaluated in silico using NCBI Primer-BLAST and then validated before quantitative analysis by endpoint PCR, followed by 1.5% agarose gel electrophoresis, which confirmed single amplicons of the predicted base-pair sizes. After each real-time qPCR run, dissociation (melting curve) analysis was performed by increasing the temperature from 60 °C to 95 °C with continuous fluorescence monitoring at 0.5 °C increments. Single, sharp melting peaks were observed for each target gene, confirming specific amplification without primer-dimers or off-target products.

To calculate absolute bacterial copy numbers, target bacterial gene fragments were amplified, purified, and quantified spectrophotometrically. The absolute copy number per microliter of standard stock was calculated using the equation:


$$\text{Copy number}/\mathrm{\mu L}=\frac{\mathrm{DNA}\;\mathrm{co}n\text{centration}\;(g/\mathrm{\mu L})\times 6.022\;x\;10^{23}}{\text{Amplicon length}\;(\mathrm{bp})\times 660\;x\;10^{9}}$$

Ten-fold serial dilutions (10^2^–10^8^ copies/μL) of purified amplicons were assayed in triplicate for each target to generate standard curves. Standard curve parameters (amplicon size, standard curve equation, *R*^2^, amplification efficiency) and amplicon sizes for each primer pair are reported in Supplementary Table S3. All assays met acceptance criteria of *R*^2^ > 0.99 and amplification efficiency between 95% and 105% (slope between −3.1 and −3.6), confirming assay linearity and reproducibility across the quantification range. Sample quantification cycle (C_q_) values were interpolated against the corresponding target-specific standard curves and normalized to express absolute abundance as log_10_ copies per gram of wet cecal digesta (28). Because qPCR analysis was restricted to five targeted bacterial groups and detects total genomic DNA, this assay evaluates quantitative shifts in the genomic abundance of these specific taxa rather than overall cecal microbial community structure, metagenomic diversity, or direct cell viability.

### Ileal cytokine gene expression analysis

Ileal tissue samples (mid-ileum) were collected from two broilers per floor pen (*n* = 24 broilers per dietary group) at slaughter (28 days of age) and preserved in an RNA-stabilizing solution (Qiagen, Germany). Total RNA was isolated from homogenized tissue (40 mg) using a commercial kit (MOLEQULE-ON, Auckland, New Zealand). RNA purity and yield were assessed spectrophotometrically using a NanoDrop 2000 (Thermo, USA), accepting samples with an A_260_/A_280_ ratio between 1.90 and 2.10 and an A_260_/A_230_ ratio >1.80. RNA integrity was further confirmed by 1% agarose gel electrophoresis showing intact 28S and 18S rRNA subunit bands. Reverse transcription was performed using 1 μg of total RNA with a commercial cDNA synthesis kit (MOLEQULE-ON, Auckland, New Zealand) in a 20 μL final reaction volume according to the following temperature program: 25 °C for 10 min, 42 °C for 50 min, and 85 °C for 5 min.

Relative expression of interleukin-6 (*IL-6*), tumor necrosis factor-alpha (*TNF-α*), interleukin-10 (*IL-10*), and interferon-gamma (*IFN-*γ) was measured in duplicate by real-time PCR (Applied Biosystems, 7,300, MA, USA) using PCR Master Mix (PCR5505, Haven Scientific, KAUST, Saudi Arabia) with target-specific primers (Supplementary Table S2). Reaction conditions were 95 °C for 5 min, followed by 40 cycles of 95 °C for 15 s and 60 °C for 30 s.

*β-actin* was evaluated as a candidate reference gene. Expression stability analysis across all experimental groups showed that *β*-actin had superior expression stability (mean Cq variation <0.3 cycles across treatment groups; *p* > 0.10; CV < 1.5%). Consequently, *β*-actin was selected as the internal reference gene for normalization. Relative fold change was calculated using the 2^−ΔΔCt^ method (29). Finally, data were averaged per pen before statistical evaluation (*n* = 12 pens per group).

### Ileal histomorphology measurements

On day 28, an unstretched 2-cm segment of the mid-ileum was collected from two birds per pen (*n* = 24 total broilers per dietary group) and fixed in 10% neutral-buffered formalin for 72 h. Tissue was processed by graded ethanol dehydration and paraffin embedding using a Tissue-Tek VIP 5 Jr automated processor (Sakura, Tokyo, Japan). Paraffin blocks were oriented to yield cross-sections perpendicular to the longitudinal axis of the intestine. One section per bird was cut (4 μm thickness) with a Leica rotary microtome (Leica Biosystems, Germany) and stained with hematoxylin and eosin (H&E).

Using a light microscope (Micros, MC500, Austria) equipped with a digital camera (100×), spatial measurements were calibrated using a 100-μm objective stage micrometer image. Ten intact, vertically oriented villi, defined as displaying a complete basement membrane and intact apical tips, were randomly selected per section. All histomorphometric measurements were performed by a trained investigator who was blinded to the dietary group identities. Anatomical landmarks for measurements were standardized as follows: villus height (μm) was measured from the top of the villus tip to the villus-crypt junction; villus width (μm) was recorded at the midpoint of the villus body; and crypt depth (μm) was measured as the distance from the base of the invagination to the villus-crypt junction. Morphometric evaluations included villus height, villus width, and crypt depth, which were used to calculate the villus height-to-crypt depth ratio and villus surface area (estimated as [villus width/2] × villus height × 6.28) (30, 31). Measurements from the two birds within each pen were averaged to yield a single statistical replicate per pen (*n* = 12 pens per group).

### Statistical analysis

For all evaluated variables, including growth performance, carcass yields, serum biochemical profiles, systemic oxidative status, mucosal cytokine expression, selected cecal bacterial DNA targets, and ileal histomorphology, the floor pen was maintained as the experimental unit (*n* = 12 pens per group). For these parameters, two broilers were sampled per pen (carcass traits, organ weights, blood biochemistry, tissue sampling, and bacterial analysis), and measurements from the two birds within each pen were averaged to generate a single pen mean before all statistical analyses, thereby consolidating within-pen subsampling variation. Individual bird values were not analyzed separately; therefore, the reported SEM reflects only between-pen variation. Before ANOVA, model assumptions were rigorously verified. Normality of residuals was evaluated using the Shapiro–Wilk test, and equality of variances was assessed using Levene’s test. When parametric assumptions were violated, data were transformed (e.g., logarithmic or square-root transformation) or analyzed using non-parametric procedures (Kruskal–Wallis test followed by *post hoc* Dunn’s test with Bonferroni adjustment). Non-parametric procedures were specifically applied to ileal cytokine relative mRNA expression (ΔΔCt) data and targeted cecal bacterial log_10_ genomic copy numbers when residual normality assumptions were not met. All reported values in the text, tables, and figures represent original, untransformed data means accompanied by their pooled standard errors of the mean (±SEM). Parametric data were analyzed by one-way analysis of variance (ANOVA) using the General Linear Model (GLM) procedure of SAS (22) according to the following linear model:

$Y_{\mathrm{ij}}=\mu +T_{i}+e_{\mathrm{ij}}.$

where Y*ij* represents the observed response parameter, μ is the overall population mean, T*i* is the fixed effect of dietary group (i = 1, …, 5), and e_ij_ is the random residual error term assumed to be normally and independently distributed.

To distinction statistically between continuous dose–response patterns and discrete mean comparisons, three hierarchical analytical steps were applied. First, the omnibus F-test was used to determine whether significant differences existed among any of the five dietary groups. When the overall F-test was significant (*p* < 0.05), Tukey’s Honestly Significant Difference (HSD) test was used to separate group means while controlling the experiment-wise Type I error rate across multiple comparisons. Linear (L) and quadratic (Q) contrast coefficients were constructed for the ordered inclusion levels (0, 1, 2, 3, 4 g/kg) to evaluate directional and nonlinear trend curves. These orthogonal contrasts test independent hypotheses regarding dose-dependent trends and are evaluated and reported separately from the omnibus *F*-test and Tukey pairwise comparisons. Statistical significance was established at *p* < 0.05, and results are presented as group means accompanied by the pooled standard error of the mean (±SEM).

## Results

### Growth performance

No mortality was recorded during the 28-day study period, resulting in 100% livability across all dietary groups. Growth indices of broilers fed different levels of defatted olive seed flour from 1 to 28 days of age are presented in Table 2. At 1 day of age, initial body weight did not differ among experimental groups (*p* = 0.996), confirming uniform weight distribution and successful randomization before dietary supplementation. During the starter period (days 1–14), broilers in the T1 and T2 groups had significantly greater body weight and body weight gain than those in the CON group (*p* < 0.05), whereas T3 and T4 did not differ significantly from CON. In addition, dietary inclusion of defatted olive seed flour (T1–T4) resulted in higher body weight and weight gain compared to the CON group during the grower phase (days 15–28) and over the full 28-day period (*p* < 0.05).

**Table 2:** Growth indices of broilers fed different levels of defatted olive seed flour (dietary groups) from 1 to 28 days of age.

Variables	Dietary groups (DG)^1^					SEM^2^	*p*-value^3^		
	CON	T1	T2	T3	T4		DG	L	Q
Body weight (g)									
1 day	43.66	43.70	43.66	43.69	43.69	0.10	0.996	0.812	0.937
14 days	506^b^	531^a^	532^a^	529^ab^	526^ab^	6.18	0.027	0.001	0.010
28 days	1629^b^	1696^a^	1724^a^	1714^a^	1716^a^	15.63	0.008	<0.0001	0.005
Weight gain (g)									
1–14 days	462^b^	488^a^	489^a^	485^ab^	482^ab^	6.15	0.027	0.001	0.011
15–28 days	1123^b^	1175^a^	1191^a^	1185^a^	1190^a^	14.71	0.011	0.009	0.052
1–28 days	1585^b^	1653^a^	1680^a^	1670^a^	1672^a^	15.60	0.007	<0.0001	0.005
Feed intake (g)									
1–14 days	553	531	538	535	538	6.07	0.117	0.011	0.073
15–28 days	1,653	1,607	1,628	1,638	1,604	15.84	0.160	0.062	0.854
1–28 days	2,207	2,138	2,166	2,173	2,141	17.61	0.061	0.012	0.448
Feed conversion ratio (g/g)									
1–14 days	1.20^a^	1.09^b^	1.10^b^	1.10^b^	1.12^b^	0.01	0.009	<0.0001	0.001
15–28 days	1.47^a^	1.38^b^	1.37^b^	1.38^b^	1.35^b^	0.02	0.001	<0.0001	0.069
1–28 days	1.39^a^	1.30^b^	1.29^b^	1.30^b^	1.28^b^	0.01	<0.0001	<0.0001	0.005
European production efficiency index									
1–14 days	252^b^	276^a^	278^a^	274^a^	279^a^	6.03	0.018	0.009	0.058
15–28 days	395^b^	440^a^	451^a^	444^a^	455^a^	9.71	0.007	<0.0001	0.023
1–28 days	417^b^	469^a^	478^a^	471^a^	479^a^	9.09	0.0001	<0.0001	0.005

^a–c^Means within the same row carrying different superscript letters are significantly different based on Tukey’s honest significant difference test (*p* < 0.05). ^1^CON = basal diet without supplementation; T1, T2, T3, and T4 = basal diet supplemented with 1, 2, 3, and 4 g defatted olive seed flour/kg of diet, respectively. ^2^Values are expressed as mean ± standard error of the mean (SEM; *n* = 12). ^3^DG = overall group effect; L = linear; Q = quadratic.

Broilers supplemented with defatted olive seed flour (T1–T4) showed an improved feed conversion ratio and a higher European Production Efficiency Index relative to the CON group (*p* < 0.05). Conversely, total feed intake was not affected by treatment over the entire experimental period (*p* > 0.05). Moreover, weight gain and feed conversion ratio during the grower phase, as well as the European production efficiency index during the starter phase, showed significant linear trends with increasing defatted olive seed flour inclusion (*p* < 0.05), consistent with the significant overall dietary group effects noted above.

Feed intake did not differ significantly among dietary groups during the starter or overall periods (*p* > 0.05), although a linear decrease was observed with increasing inclusion levels (*p* < 0.05). Furthermore, significant quadratic responses (*p* < 0.05) were observed for body weight (14 and 28 days; weight gain and feed conversion ratio during the starter phase and over the full 28-day period; and the production efficiency index during the grower phase and over the full 28-day period (*p* < 0.05).

### Carcass traits, relative organ weights, and breast meat physical quality

Carcass characteristics, relative internal organ weights, and breast meat physical quality traits of broilers fed different levels of defatted olive seed flour are presented in Table 3. Carcass weight and dressing yield increased in broilers receiving T3 and T4 than CON group (*p* < 0.05), while there was no significant difference between T1 and T2. In addition, there was a linear response to the different levels of defatted olive seed flour (*p* < 0.05). Slaughter weight and the relative weights of legs and breast showed no effect in any dietary group and no linear or quadratic response (*p* > 0.05).

**Table 3:** Carcass traits, internal organ, and breast meat physical quality of broilers fed different levels of defatted olive seed flour (dietary groups) at 28 days of age.

Variables	Dietary groups (DG)^1^					SEM^2^	*p*-value^3^		
	CON	T1	T2	T3	T4		DG	L	Q
Carcass traits									
Slaughter weight (g)	1,591	1,687	1,656	1,664	1,675	36.33	0.390	0.058	0.337
Carcass weight (g)	1011^b^	1083^ab^	1103^ab^	1129^a^	1118^a^	25.63	0.019	0.001	0.093
Dressing yield (%)	63.53^b^	64.22^b^	66.59^ab^	68.02^a^	66.77^a^	0.92	0.007	0.008	0.174
Legs (%)	24.88	25.91	25.77	25.97	26.14	0.87	0.865	0.285	0.670
Breast (%)	26.84	26.90	27.92	25.68	26.85	1.05	0.692	0.999	0.795
Relative internal organ weight (%)									
Thymus	0.23^b^	0.52^a^	0.43^a^	0.45^a^	0.44^a^	0.04	0.003	<0.0001	0.006
Bursa of fabricius	0.20^b^	0.32^a^	0.26^ab^	0.27^ab^	0.26^ab^	0.01	0.003	0.002	0.018
Spleen	0.09	0.14	0.14	0.18	0.13	0.02	0.172	0.092	0.093
Liver	2.34	2.30	2.32	2.26	2.26	0.08	0.951	0.594	0.941
Intestine	4.59	5.21	4.77	4.87	4.72	0.19	0.233	0.172	0.180
Breast meat physical traits									
pH	5.62	5.52	5.84	5.69	5.79	0.12	0.376	0.602	0.942
Lightness	51.24	54.04	54.04	54.32	53.41	1.22	0.388	0.054	0.117
Redness	2.67	2.28	3.08	3.00	2.86	0.28	0.323	0.677	0.725
Yellowness	13.45	13.96	14.23	14.65	13.18	0.59	0.419	0.411	0.089

^a–b^Means within the same row carrying different superscript letters are significantly different based on Tukey’s honest significant difference test (*p* < 0.05). ^1^CON = basal diet without supplementation; T1, T2, T3, and T4 = basal diet supplemented with 1, 2, 3, and 4 g defatted olive seed flour/kg of diet, respectively. ^2^Values are expressed as mean ± standard error of the mean (SEM; *n* = 12). ^3^DG = overall group effect; L = linear; Q = quadratic.

Dietary inclusion of defatted olive seed flour (T1–T4) increased the relative weight of the thymus compared with the CON group (*p* < 0.05). Broilers receiving T1 had a higher relative weight of the bursa of Fabricius than the CON group (*p* < 0.05), but there was no significant difference in the other dietary groups. A quadratic response in the relative weights of the thymus and bursa of Fabricius was observed with dietary inclusion of defatted olive seed flour (*p* < 0.05). Furthermore, the relative weights of other internal organs (spleen, liver, and intestine) and breast meat physical traits (pH, lightness, redness, and yellowness) showed no effect in any dietary group and no linear or quadratic response (*p* > 0.05).

### Serum biochemical profile and oxidative status

Serum biochemical profile and oxidative status biomarkers of broilers fed different levels of defatted olive seed flour are presented in Table 4. Concentrations of total protein and globulin increased in broilers receiving T2 compared with CON (*p* < 0.05), while there was no significant difference compared with T1, T3, and T4. In addition, there was a quadratic response to the different levels of defatted olive seed flour (*p* < 0.05). Broilers receiving T4 had a lower concentration of total cholesterol than the CON, T1, and T2 groups (*p* < 0.05), but no significant difference compared with T3. A linear response of total cholesterol concentration was observed with dietary inclusion of defatted olive seed flour (*p* < 0.05).

**Table 4:** Serum biochemical profile and oxidative status biomarkers of broilers fed different dietary levels of defatted olive seed flour (dietary groups) at 28 days of age.

Variables	Dietary groups (DG)^1^					SEM^2^	*p*-value^3^		
	CON	T1	T2	T3	T4		DG	L	Q
Serum biochemistry									
Total protein, g/dL	3.25^b^	3.70^ab^	3.89^a^	3.68^ab^	3.50^ab^	0.13	0.029	0.005	0.003
Albumin, g/dL	1.84	1.91	1.86	1.90	1.66	0.07	0.243	0.916	0.082
Globulin, g/dL	1.41^b^	1.79^ab^	2.03^a^	1.79^ab^	1.84^ab^	0.12	0.019	0.001	0.026
Glucose, mg/dL	177	155	155	163	153	5.94	0.053	0.004	0.204
Total cholesterol, mg/dL	156.39^a^	146.32^ab^	146.69^ab^	136.84^bc^	121.20^c^	4.32	<0.0001	0.005	0.233
Triglycerides, mg/dL	101.35^a^	74.01^b^	70.75^b^	92.18^a^	98.47^a^	2.92	<0.0001	<0.0001	<0.0001
HDL-cholesterol, mg/dL	50.76^b^	84.33^a^	77.76^a^	52.64^b^	46.34^b^	5.79	0.001	0.033	0.004
LDL-cholesterol, mg/dL	85.35^a^	47.20^b^	54.78^b^	65.77^ab^	55.17^ab^	7.06	0.007	0.007	0.061
Uric acid, mg/dL	5.81^ab^	5.76^ab^	5.51^ab^	6.16^a^	5.36^b^	0.16	0.023	0.554	0.362
Creatinine, mg/dL	0.961^a^	0.887^ab^	0.837^ab^	0.891^ab^	0.737^b^	0.04	0.040	0.025	0.741
Aspartate aminotransferase, U/L	212	205	196	207	195	4.99	0.138	0.053	0.623
Oxidative status biomarkers									
Total antioxidant capacity, μmol/mL	15.14^b^	15.65^b^	16.77^a^	17.00^a^	16.73^a^	0.25	<0.0001	<0.0001	0.016
Malondialdehyde, nmol/mL	6.10^a^	5.97^a^	3.83^b^	3.86^b^	4.35^b^	0.50	0.003	0.010	0.045

^a–c^Means within the same row carrying different superscript letters are significantly different based on Tukey’s honest significant difference test (*p* < 0.05). ^1^CON = basal diet without supplementation; T1, T2, T3, and T4 = basal diet supplemented with 1, 2, 3, and 4 g defatted olive seed flour/kg of diet, respectively. ^2^Values are expressed as mean ± standard error of the mean (SEM; *n* = 12). ^3^DG = overall group effect; L = linear; Q = quadratic.

Dietary inclusion of defatted olive seed flour (T1 and T2) decreased triglycerides and increased HDL-cholesterol compared with the other dietary groups (*p* < 0.05). In addition, there was a quadratic response to the different levels of defatted olive seed flour (*p* < 0.05). Broilers receiving T1 and T2 had lower LDL-cholesterol concentrations than the CON group (*p* < 0.05), but no significant difference compared with T3 and T4. A linear response of LDL-cholesterol was observed with dietary inclusion of defatted olive seed flour (*p* < 0.05). Furthermore, broilers receiving T4 had lower uric acid concentrations than T3 group (*p* < 0.05), but no significant difference compared with other groups.

The concentration of creatinine decreased in broilers receiving T4 compared with CON (*p* < 0.05), with no significant differences from the other dietary groups. Moreover, there was a linear response to the different levels of defatted olive seed flour (*p* < 0.05). Albumin and aspartate aminotransferase showed no significant overall dietary group effect and no linear or quadratic trend (*p* > 0.05). Glucose concentration likewise showed no significant overall dietary group effect (*p* = 0.053); however, a significant linear trend toward lower glucose concentrations was detected with increasing defatted olive seed flour inclusion (*p* = 0.004), which was not accompanied by a significant quadratic trend (*p* = 0.204). For oxidative status biomarkers, broilers receiving 2, 3, and 4 g defatted olive seed flour/kg of diet (T2–T4) had increased total antioxidant capacity and decreased malondialdehyde concentration compared with the CON and T1 groups (*p* < 0.05), and a quadratic response was observed among dietary groups (*p* < 0.05).

### Ileal cytokine gene expression

Ileal cytokine gene expression of broilers fed different levels of defatted olive seed flour is presented in Figure 1. Broilers receiving CON had a higher relative fold change in *IL-6* and *TNF-α* mRNA expression compared with the T3 group (*p* < 0.05), but no significant difference compared with the other dietary groups. Relative fold change in *IL-10* transcript abundance increased in broilers receiving T3, followed by T2 and T4, compared with the CON and T1 groups (*p* < 0.05). In addition, dietary inclusion of defatted olive seed flour (T2–T4) increased the fold change in *IFN-*γ expression compared with the CON group (*p* < 0.05), but showed no significant difference compared with T1. Moreover, a quadratic response of all ileal cytokine gene expressions (*IL-6*, *TNF-α*, *IL-10*, and *IFN-*γ) was observed with dietary inclusion of defatted olive seed flour (*p* < 0.05).

**Figure 1:**
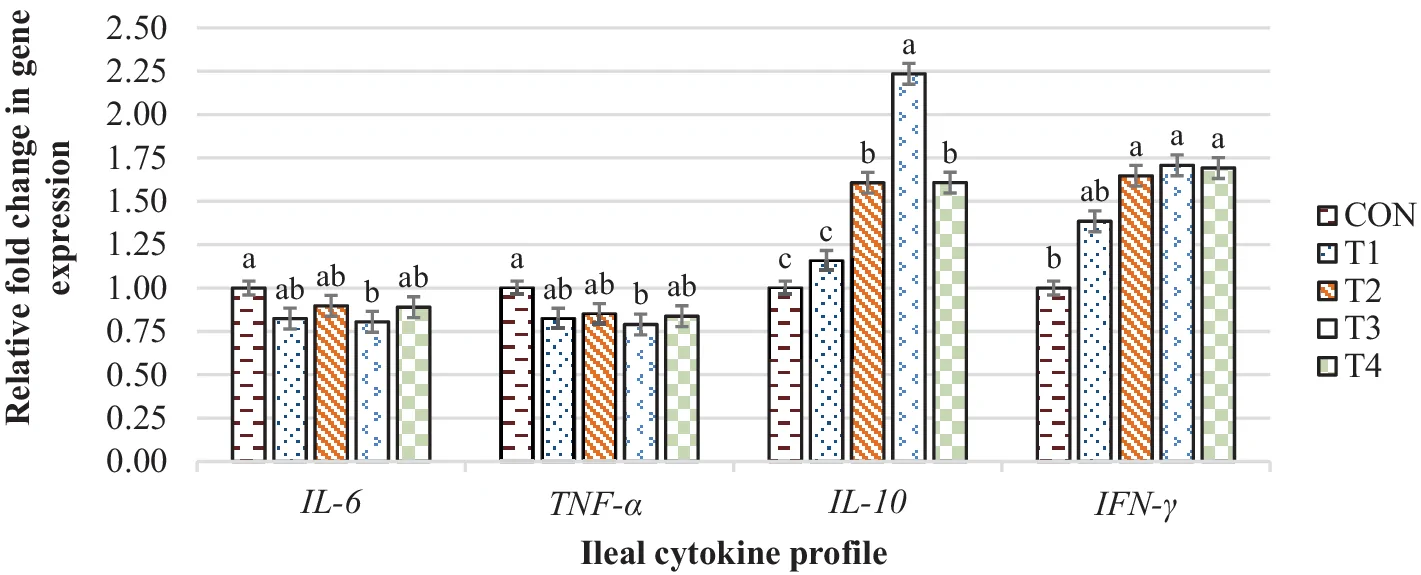
Ileal cytokine gene expression of broilers fed different dietary levels of defatted olive seed flour (dietary groups 1) at 28 days of age. ^a–c^Means within the same column carrying different superscript letters are significantly different based on Tukey’s honest significant difference test (*p* < 0.05). ^1^CON = basal diet without supplementation; T1, T2, T3, and T4 = basal diet supplemented with 1, 2, 3, and 4 g defatted olive seed flour/kg of diet, respectively. Values are expressed as mean ± standard error of the mean (SEM; *n* = 12). Error bars represent the pooled SEM; values are fold changes relative to CON (2^−ΔΔCt^ method, normalized to *β-actin*). *p*-value: *IL-6* = interleukin-6 (overall group effect = 0.040; linear = 0.006; quadratic = 0.049), *TNF-α* = tumor necrosis factor-alpha (overall group effect = 0.020; linear = 0.001; quadratic = 0.032), *IL-10* = interleukin-10 (overall group effect = <0.0001; linear = <0.0001; quadratic = 0.008), and *IFN-*γ = interferon-gamma (overall group effect = <0.0001; linear = <0.0001; quadratic = 0.006).

### Absolute cecal selected bacterial DNA targets

Absolute cecal bacterial DNA target abundances of broilers fed different levels of defatted olive seed flour are presented in Figure 2. Dietary inclusion of defatted olive seed flour (T1–T4) increased quantitative *Lactobacillus* spp. copy numbers compared with the CON group (*p* < 0.05). In contrast, quantitative *Bifidobacterium* spp. DNA abundance showed no differences among dietary groups and no linear or quadratic responses (*p* > 0.05). Broilers receiving T3 had lower quantitative *Salmonella* spp. copy numbers compared with the CON and T1 groups (*p* < 0.05), with no significant differences compared with the T2 and T4 groups. Quantitative *Clostridium* spp. and *Escherichia coli* DNA target abundances decreased in broilers receiving defatted olive seed flour (T1–T4) compared with the CON group (*p* < 0.05). Moreover, quadratic responses of *Lactobacillus* spp., *Salmonella* spp., *Clostridium* spp., and *Escherichia coli* were observed with dietary inclusion of defatted olive seed flour (*p* < 0.05).

**Figure 2:**
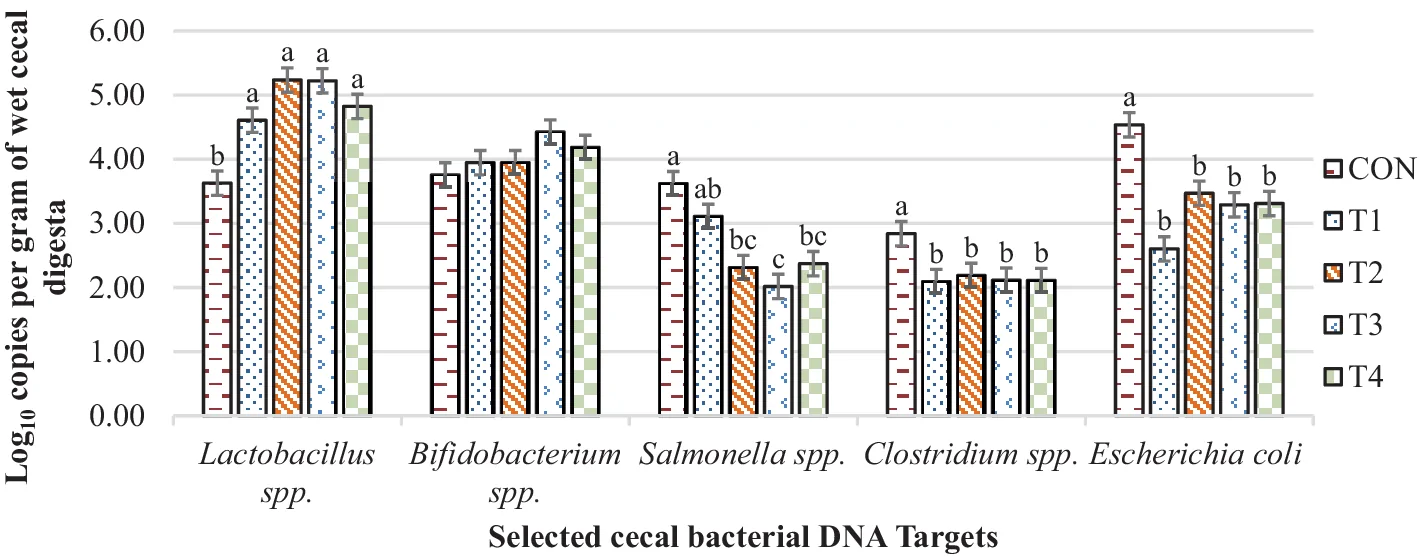
Absolute abundance of selected caecal bacterial DNA targets in broilers fed different dietary levels of defatted olive seed flour (dietary groups 1) at 28 days of age. ^a–c^Means within the same column carrying different superscript letters are significantly different based on Tukey’s honest significant difference test (*p* < 0.05). ^1^CON = basal diet without supplementation; T1, T2, T3, and T4 = basal diet supplemented with 1, 2, 3, and 4 g defatted olive seed flour/kg of diet, respectively. Values are expressed as mean ± standard error of the mean (SEM; *n* = 12). Error bars represent the pooled SEM; abundance is expressed as log_10_ copies per gram of wet cecal digesta. *p*-value: *Lactobacillus* spp. (overall group effect = <0.0001; linear = <0.0001; quadratic = <0.0001), *Bifidobacterium* spp. (overall group effect = 0.158; linear = 0.098; quadratic = 0.606), *Salmonella* spp. (overall group effect = 0.006; linear = 0.003; quadratic 0.018), *Clostridium* spp. (overall group effect = 0.001; linear = <0.0001; quadratic = 0.016), and *Escherichia coli* (overall group effect = 0.002; linear = <0.0001; quadratic = 0.021).

### Ileal histomorphology

Ileal histomorphology measurements of broilers fed different levels of defatted olive seed flour are presented in Table 5. Broilers receiving T3 had greater villus height and surface area than the other groups (*p* < 0.05). Villus width increased in broilers receiving defatted olive seed flour (T2–T4) compared with the CON and T1 groups (*p* < 0.05). In addition, broilers receiving T3 and T4 had greater crypt depth than the T1 and T2 groups, but lower crypt depth than the CON group (*p* < 0.05). Moreover, a quadratic response in villus height, width, and surface area was observed with dietary inclusion of defatted olive seed flour (*p* < 0.05). In contrast, there was a linear response for crypt depth (*p* < 0.05). The villus height-to-crypt depth ratio showed no differences among dietary groups and no linear or quadratic response (*p* > 0.05).

**Table 5:** Ileal histomorphology measurements of broilers fed different dietary levels of defatted olive seed flour (dietary groups) at 28 days of age.

Variables	Dietary groups (DG)^1^					SEM^2^	*p*-value^3^		
	CON	T1	T2	T3	T4		DG	L	Q
Villus height (μm)	788.30^b^	810.24^b^	829.00^b^	936.19^a^	822.43^b^	18.51	<0.0001	0.003	0.015
Villus width (μm)	76.82^b^	90.55^ab^	95.21^a^	100.52^a^	94.05^a^	4.08	0.001	0.001	0.010
Villus surface area (mm^2^)	0.19^c^	0.23^bc^	0.25^b^	0.29^a^	0.24^b^	0.01	<0.0001	<0.0001	0.007
Crypt depth (μm)	82.08^a^	60.96^c^	66.32^c^	76.70^b^	70.93^b^	1.97	<0.0001	0.003	0.582
Villus height-to-crypt depth ratio	13.13	13.65	12.82	12.35	11.75	0.48	0.071	0.374	0.304

^a–c^Means within the same row carrying different superscript letters are significantly different based on Tukey’s honest significant difference test (*p* < 0.05). ^1^CON = basal diet without supplementation; T1, T2, T3, and T4 = basal diet supplemented with 1, 2, 3, and 4 g defatted olive seed flour/kg of diet, respectively. ^2^Values are expressed as mean ± standard error of the mean (SEM; *n* = 12). ^3^DG = overall group effect; L = linear; Q = quadratic.

## Discussion

The search for sustainable, functional feed ingredients that enhance broiler growth performance, gut health, and oxidative capacity while replacing traditional antibiotic growth promoters has focused attention on agricultural by-products (15). Olive oil extraction yields substantial secondary materials rich in natural bioactive compounds (8). Defatted olive seed flour is a particularly valuable resource in this context. Mechanical cold-pressing followed by solvent extraction produces a matrix concentrated in crude fiber (30.0%) and functional polyphenols while removing excess triacylglycerols (16). In our previous study, gas chromatography–mass spectrometry confirmed the rich bioactive composition of defatted olive seed flour, revealing a predominantly monounsaturated fatty acid profile along with key secondary metabolites, including hydroxytyrosol, oleuropein, and squalene (17). Although these phenolics and terpenes are recognized in the literature for their radical-scavenging and mucosal-protective properties in broilers (32, 33), the present study evaluated the total defatted olive seed flour matrix. Therefore, the observed physiological effects reflect the synergistic contribution of the entire dietary additive rather than the actions of the individually quantified compounds.

In the broader literature, the nutritional efficacy of olive-derived co-products in poultry diets varies considerably with the processing method, inclusion level, and physical form. Several studies report positive responses to extracted polyphenols or fermented pomace (12, 14), whereas feeding raw, unrefined olive cake or crude pomace frequently yields neutral or even negative effects on broiler growth, feed conversion, and carcass yield because of elevated crude fiber, high lignin content, and residual lipid oxidation (34, 35). For example, higher inclusion levels of raw olive cake or leaf meal (>20 g/kg) often fail to improve body weight gain or feed conversion efficiency and may reduce dry matter digestibility due to gastrointestinal anti-nutritional factors (36, 37). In contrast, the low inclusion rates (1–4 g/kg) and standardized processing of defatted olive seed flour used in the present trial may have limited these high-fiber, anti-nutritional drawbacks, although digestibility was not measured; responses were observed without a reduction in feed intake. These findings build on the growing body of literature evaluating olive industry by-products (14, 25, 38) by demonstrating the baseline efficacy of a refined defatted seed matrix across an expanded gradient (1–4 g/kg feed) in Ross 308 broilers over a 28-day trial.

Dietary supplementation with defatted olive seed flour was associated with significantly better growth indices, specifically final body weight, total weight gain, feed conversion ratio, and production efficiency index, without compromising feed intake. Quadratic and linear responses were observed, with low-to-moderate levels (1–2 g/kg) optimizing early starter growth, whereas all inclusion levels (1–4 g/kg) conferred significant performance benefits during the grower and overall stages. Because feed intake was preserved across groups, inclusion of defatted olive seed flour up to 4 g/kg evidently did not impair diet palatability. The improved weight gain and feed efficiency were observed alongside altered ileal morphology in birds fed diets containing the fiber and phenolic compounds of defatted olive seed flour; nutrient digestibility and absorption were not measured, so any link between these endpoints remains associative. These findings align with those of Herrero-Encinas et al. (12), who reported improved feed efficiency and growth in broilers receiving olive pomace extracts. However, our findings contrast with reports in which unrefined olive by-products had neutral effects on growth rate (35–37), highlighting that low-dose inclusion of refined defatted seed flour behaves differently from raw, high-fiber olive residues. Ferlisi et al. (39) likewise found that olive-derived polyphenols support nutrient absorption by improving the structural architecture of the intestinal mucosa.

An interesting finding in the present study was that carcass weight and dressing yield increased significantly and linearly, peaking at higher inclusion rates (3–4 g/kg), even though preslaughter live body weight remained statistically unchanged across groups. This increase did not affect primal cut yields (breast and leg percentages) or meat quality parameters (pH and color coordinates). This finding indicates that carcass weight and dressing yield were higher with defatted olive seed flour inclusion without a detectable change in slaughter weight; nutrient partitioning, tissue accretion, and nutrient retention were not measured. Possible explanations, drawn from previous literature rather than from measurements in this study, are that polyphenols and fibers may support gut mucosal architecture and shift the abundance of targeted cecal bacterial taxa, which could in turn influence nutrient utilization (15, 17, 19). Phytogenic and fiber components may also alter gut transit or luminal content weight and thereby increase the eviscerated carcass proportion; however, the relative weights of the intestine and liver did not differ among groups in this trial, so this explanation remains speculative. The relative weights of primary lymphoid organs, specifically the thymus and bursa of Fabricius, showed a quadratic response across dietary inclusion levels. Although relative organ mass provides information on organ development, variation in organ weight alone does not directly indicate changes in systemic immunocompetence or functional immune capacity (40). In addition, the bursal weight responses observed in this study occurred against the backdrop of the routine infectious bursal disease vaccination administered during the trial, which itself influences bursal morphometrics and developmental dynamics. Notably, the relative weights of the liver and spleen remained unchanged across groups, indicating that the observed variations were specific to the primary lymphoid tissues and were not accompanied by systemic organomegaly.

Serum biochemical markers provide insight into protein metabolism, hepatic function, and lipid homeostasis (41). At an inclusion rate of 2 g/kg, defatted olive seed flour was associated with higher circulating total protein and globulin concentrations, reflecting shifts in the protein metabolic profile and circulating protein fractions. Similar elevations in serum globulins following dietary administration of olive by-products were reported by Negm et al. (15), which may indicate immunomodulation, although immunoglobulins and immune function were not measured directly. A prominent physiological effect observed in the present study was the modulation of serum lipid profiles: total cholesterol showed a dose-dependent linear decrease that peaked at 4 g defatted olive seed flour/kg, whereas lower to moderate dietary concentrations (1 and 2 g/kg) significantly modulated circulating lipid fractions by lowering triglycerides and increasing HDL-cholesterol. These shifts demonstrate target-specific alterations in circulating lipid fractions rather than uniform suppression across all lipid parameters. While olive bioactive compounds and unsaturated fatty acid residues, are frequently associated with altered lipid transport (42) and metabolism, hepatic enzyme activity and lipid synthesis pathways were not directly evaluated in this study. Further biochemical work is required to determine the specific metabolic mechanisms underlying these serum lipid responses. Furthermore, renal function screening markers were maintained or improved: creatinine decreased linearly with defatted olive seed flour inclusion, and uric acid was lowest at 4 g defatted olive seed flour/kg, indicating that no adverse changes occurred in the serum biochemical markers of hepatic and renal function evaluated.

In intensive broiler production, rapid metabolic growth generates endogenous reactive oxygen species, leading to oxidative stress and lipid peroxidation (43). Serum oxidative biomarkers showed that including defatted olive seed flour at 2–4 g/kg increased total antioxidant capacity while reducing malondialdehyde concentrations. Because malondialdehyde is a primary biomarker of lipid peroxidation, its lower concentration is consistent with reduced lipid peroxidation (44). The higher serum total antioxidant capacity and concurrent reduction in malondialdehyde observed with defatted olive seed flour supplementation are consistent with the dietary inclusion of olive phenolics and other bioactive compounds. Although previous literature suggests that olive-derived polyphenols may stimulate endogenous cellular defenses through the Nrf2 signaling pathway or via upregulation of superoxide dismutase and glutathione peroxidase (18), these molecular pathways and downstream antioxidant enzyme activities were not directly measured in the present study. Therefore, although our data show higher serum antioxidant capacity and lower lipid peroxidation, any mechanistic link to specific intracellular signaling cascades remains a hypothesis derived from existing literature rather than an empirically confirmed finding of this trial. These results agree with Ibrahim et al. (14) and Erinle et al. (45), who reported reduced malondialdehyde accumulation and enhanced systemic antioxidant defenses in broilers fed olive pomace derivatives.

The structural architecture of the intestinal mucosa provides clues about potential digestive and absorptive capacity (46). In the current study, inclusion of defatted olive seed flour at 3 g/kg was associated with the greatest villus height and surface area. These histomorphometric changes increase the physical surface area of the mucosa and may be associated with enhanced intestinal absorptive capacity, which coincided with the observed improvements in feed conversion ratio and growth performance. Although crypt depth increased at higher inclusion levels (3–4 g/kg), the overall villus height-to-crypt depth ratio remained stable, reflecting structural balance in mucosal dimensions without overt morphological damage. Intestinal mucosal immunity plays a pivotal role in maintaining gut homeostasis (47). Analysis of ileal cytokine mRNA levels showed quadratic responses across all evaluated target genes (*IL-6*, *TNF-α*, *IL-10*, and *IFN-*γ). Supplementation with defatted olive seed flour at 3 g/kg feed was associated with higher *IL-10* and *IFN-*γ transcript abundance and lower *IL-6* and *TNF-α* mRNA expression relative to the control group. *IL-10* acts as a crucial regulatory cytokine that prevents excessive inflammatory damage in the gut epithelium of broilers (48). In contrast, *IFN-*γ is generally regarded as a pro-inflammatory (Th1-type) cytokine, so the concurrent rise in *IL-10* and *IFN-*γ with lower *IL-6* and *TNF-α* reflects a mixed transcript profile that cannot be interpreted as uniformly anti-inflammatory. Because relative gene expression measured by RT-qPCR reflects transcriptional activity rather than translated protein abundance or functional cellular immunity, these changes indicate local transcriptional modulation within the ileal mucosa rather than definitive optimization of systemic functional immunity (19).

The cecal bacterial community maintains competitive exclusion against non-beneficial microorganisms and produces short-chain fatty acids that nourish the intestinal mucosa (49). Real-time PCR quantification showed that defatted olive seed flour supplementation (1–4 g/kg) was associated with higher *Lactobacillus* spp. copy numbers and lower abundance of DNA targets for *Clostridium* spp., *Escherichia coli*, and *Salmonella* spp. *Bifidobacterium* spp. remained consistent across groups. Because only five bacterial groups were quantified by qPCR, these observed shifts reflect targeted changes in specific bacterial populations rather than comprehensive alterations in overall microbial community structure or a generalized prebiotic effect. These shifts in absolute abundance of selected caecal bacterial DNA targets coincided with the presence of crude fiber (30.0%) and polyphenolic compounds in defatted olive seed flour (16). Non-starch polysaccharides serve as fermentable substrates for lactic acid-producing bacteria such as *Lactobacillus* spp. The resulting lactic and acetic acids could acidify the cecal lumen, potentially creating an unfavorable microenvironment for acid-sensitive organisms, including *Escherichia coli*, *Salmonella* spp., and specific *Clostridium species* (50). Cecal pH and short-chain fatty acids were not measured in this study, so this pathway remains hypothetical. Beyond this indirect microbial exclusion, polyphenols from olive products have been reported *in vitro* to exert antibacterial action by compromising bacterial cell membrane permeability (51); whether this occurred in the gut of the birds in the present study was not tested. These microbiological findings align with recent evidence showing that targeted nutritional interventions and functional feed additives can modulate gut microbiota composition and support intestinal mucosal integrity, which has been linked to improved gut health (52–54).

Several limitations should be considered when interpreting these findings. The trial involved male Ross 308 broilers reared under controlled conditions for 28 days; therefore, the results require confirmation in other strains, mixed-sex flocks, and commercial production conditions. In addition, because cecal analysis was restricted to five specific bacterial taxa quantified by real-time PCR, this targeted approach cannot represent overall microbial community structure, beta diversity, or high-throughput metagenomic profile shifts. Furthermore, standard qPCR detects total bacterial genomic DNA abundance without differentiating between viable and nonviable bacterial cells, meaning target copy numbers do not directly reflect metabolically active or viable cell counts. Future studies incorporating 16S rRNA gene amplicon sequencing and viability-based approaches (such as viability PCR or culture-based quantification) are needed to evaluate broader, community-wide microbiome dynamics and functional viability. The mechanisms proposed to explain the antioxidant, metabolic, and inflammatory responses were inferred from the measured endpoints and previous studies rather than tested directly. Nutrient digestibility, cecal pH and short-chain fatty acids, antioxidant enzyme activities, and cytokine protein levels were not measured. Further work should evaluate defatted olive seed flour under commercial or challenge conditions, apply broader microbiome approaches, and assess its economic feasibility and effects on meat quality during storage.

## Conclusion

In conclusion, dietary supplementation with defatted olive seed flour was associated with positive shifts in broiler growth performance, targeted cecal bacterial DNA abundance, and systemic antioxidant status under the specific experimental conditions of this 28-day trial in male Ross 308 broilers. Dietary inclusion across all tested levels (1–4 g/kg feed) was consistently associated with enhanced body weight gain, feed conversion efficiency, and the European Production Efficiency Index without suppressing feed intake. Higher inclusion rates (3–4 g/kg feed) were associated with increased carcass weight and dressing yield. Furthermore, low-to-moderate supplementation (1–2 g/kg feed) was associated with lower serum triglycerides and LDL cholesterol, whereas inclusion levels between 2 and 4 g/kg were associated with elevated serum total antioxidant capacity and lowered lipid peroxidation (malondialdehyde).

Within the gastrointestinal tract, supplementation with defatted olive seed flour was associated with an increased quantitative abundance of cecal *Lactobacillus* spp. DNA and lower copy numbers of *Clostridium* spp., *Escherichia coli*, and *Salmonella* spp. targets. These cecal shifts were accompanied by altered ileal mucosal histomorphology (greater villus height and surface area at 3 g/kg) and concurrent transcriptional modulation of ileal cytokine transcripts (increased *IL-10* and *IFN-*γ transcripts at 3 g/kg). Because responses differed among the measured outcomes, no single inclusion level uniformly optimized all measured traits. Because responses differed among the measured outcomes, no single inclusion level uniformly optimized all measured traits. Responses across the measured endpoints were generally favouable at 2–3 g/kg, although no single inclusion level consistently improved all outcomes under the conditions of the present study.

## Data Availability

The data that support the findings of this study are available from the corresponding author upon reasonable request.
