# Supplementary material for: Effects of dietary defatted olive seed flour supplementation on growth performance, systemic status, and intestinal health in broiler chickens
Source: Front Vet Sci. 2026 Oct 21;13:2002766. doi: 10.3389/fvets.2026.2002766 (PMC13731863; doi:10.3389/fvets.2026.2002766)
Supplement: Supplementary file 1 [file Table_1.docx]

**Table S1.** Chemical characterization of defatted olive seed flour determined by gas chromatography–mass spectrometry (GC-MS).

| Chemical Category | Compound Name | Retention Time (min) | Relative Area (%) |
| --- | --- | --- | --- |
| Fatty acids and esters | Oleic acid | 30.04 | 82.93 |
|  | Palmitic acid | 27.15 | 9.09 |
|  | Stearic acid | 30.26 | 3.56 |
|  | Palmitoleic acid | 29.32 | 1.46 |
|  | Oleyl alcohol, trifluoroacetate | 26.81 | 0.44 |
|  | Hexadecanoic acid, methyl ester | 26.54 | 0.31 |
|  | 9,12-Octadecadienoic acid (Z,Z)- | 29.24 | 0.23 |
|  | Linoleic acid | 28.37 | 0.11 |
|  | Ascorbyl dipalmitate | 27.53 | 0.04 |
|  | Margaric acid | 28.68 | 0.07 |
|  | Pentadecanoic acid | 28.91 | 0.02 |
| Bioactive phenolics and terpenes | Oleuropein | 9.90 | 0.49 |
|  | Squalene | 38.90 | 0.47 |
|  | Hydroxytyrosol | 17.42 | 0.13 |
| Hydrocarbons and volatiles | 2,2-Dimethoxybutane | 3.37 | 0.18 |
|  | Heptadecene | 20.59 | 0.10 |
|  | Pentadecene | 16.52 | 0.10 |
|  | Dodecane | 12.22 | 0.09 |
|  | Carbonic acid ester | 24.26 | 0.07 |
|  | Cyclopentasiloxane, decamethyl- | 11.09 | 0.06 |
|  | Silane, dimethoxymethylphenyl- | 11.25 | 0.06 |
| Total identified | |  | 100.00 |
| Note: Values represent the relative peak area percentage (%) calculated as (individual peak area / total integrated chromatogram area) × 100. Relative area percentages indicate the proportion of each compound within the GC–MS response and do not reflect absolute quantitative mass concentrations due to variations in compound response factors and the absence of external calibration standards | | | |

**Table S2.** Oligonucleotide primer sequences and corresponding GenBank accession numbers used for real-time PCR quantification of ileal cytokine and reference genes and cecal bacterial DNA targets.

| **Target gene** | **Primer sequences (5′ → 3′)** | **Product**  **length**  **(bp)** | **Annealing temperature (℃)** | **GenBank accession**  **number** |
| --- | --- | --- | --- | --- |
| Host immune and reference genes | | | | |
| *IL-6* | F: GGAGTGGCCAAGAACCAAGA  R: ATGCTAAGGCACAGCACACT | 109 | 60 | AB028635.1 |
| *TNF-α* | F: GGGAGTGTGAGGGGTATCCT  R: CTGCACCTTCTGTCTCGGTT | 93 | 60 | [MH180383.1](https://www.ncbi.nlm.nih.gov/nuccore/MH180383.1) |
| *IL-10* | F: ACCGTGATTGGCAGGTACAG  R: CTGGCCCTGGGTTCACTTAG | 123 | 58 | JQ687536.1 |
| *INF-*γ | F: TCCCAGAAGCTATCTGAGCAT  R: CCACCGTCAGCTACATCTGAAT | 200 | 58 | [NM_205149.2](https://www.ncbi.nlm.nih.gov/nuccore/NM_205149.2) |
| *β-actin* | F: CCTTCCTGGGTAGGTGTCG  R: TGGCGTAGAGGTCCTTCCTG | 188 | 60 | [AJ312193.1](https://www.ncbi.nlm.nih.gov/nuccore/AJ312193.1) |
| Cecal bacterial DNA targets | | | | |
| *Lactobacillus spp.* | F: CGACTGCTCTGGTTATACCGT  R: TGAAGAAGGGTTTCGGCTCG | 93 | 60 | NR_040806.1 |
| *Bifidobacterium spp.* | F: CAGCTCGTGTCGTGAGATGT  R: GATCTGACGTCATCCCCACC | 136 | 59 | NR_043433.1 |
| *Salmonella spp.* | F: TCATCACCACCAACAGTAC  R: ACTGACTGAACACGGAAG | 71 | 58 | NR_074900.1 |
| *Clostridium spp.* | F: GTTTACGGCGTGGACTACCA  R: TGGAAGTCTAGAGTGCGGGA | 174 | 58 | NR_114788.1 |
| *Escherichia coli* | F: CATGCCGCGTGTATGAAGA  R: GGGTAACGTCAATGACAAAG | 95 | 60 | NR_024570.1 |

Primers were designed using Primer-BLAST (NCBI) to ensure target specificity and prevent cross-reactivity with non-target sequences. Specificity was further verified by endpoint PCR with agarose gel electrophoresis (single amplicon of the expected size) and by melting-curve analysis (single peak) for each target, as described in the Materials and Methods.

**Table S3.** Real-time PCR assay performance parameters, amplicon characteristics, and standard curve metrics.

| Target gene | Amplicon Size (bp) | Standard Curve Equation | Efficiency  (E, %) | Linearity  (R^2^) |
| --- | --- | --- | --- | --- |
| *Lactobacillus spp.* | 186 | y = -3.332x + 37.10 | 99.6 | 0.997 |
| *Bifidobacterium spp.* | 210 | y = -3.368x + 36.82 | 98.1 | 0.995 |
| *Salmonella spp.* | 142 | y = -3.425x + 38.05 | 95.9 | 0.993 |
| *Clostridium spp.* | 195 | y = -3.295x + 37.40 | 101.1 | 0.996 |
| *Escherichia coli* | 178 | y = -3.401x + 37.92 | 96.8 | 0.994 |
